# An Analysis of the Structural Relationship between Thyroid Hormone-Signaling Disruption and Polybrominated Diphenyl Ethers: Potential Implications for Male Infertility

**DOI:** 10.3390/ijms24043296

**Published:** 2023-02-07

**Authors:** Ishfaq Ahmad Sheikh, Mohd Amin Beg, Taha Abo-Almagd Abdel-Meguid Hamoda, Hammam Mahmoud Siraj Mandourah, Erdogan Memili

**Affiliations:** 1 King Fahd Medical Research Center, King Abdulaziz University, Jeddah 21589, Saudi Arabia; 2 Department of Medical Laboratory Sciences, Faculty of Applied Medical Sciences, King Abdulaziz University, Jeddah 21589, Saudi Arabia; 3 Department of Urology, Faculty of Medicine, King Abdulaziz University, Jeddah 21589, Saudi Arabia; 4 College of Agriculture and Human Sciences, Prairie View A&M University, Prairie View, TX 77446, USA

**Keywords:** endocrine disruption, flame retardants, polybrominated diphenyl ethers, structural studies, thyroid hormone receptor, thyroid signaling dysfunction

## Abstract

Polybrominated diphenyl ethers (PBDEs) are a common class of anthropogenic organobromine chemicals with fire-retardant properties and are extensively used in consumer products, such as electrical and electronic equipment, furniture, textiles, and foams. Due to their extensive use, PBDEs have wide eco-chemical dissemination and tend to bioaccumulate in wildlife and humans with many potential adverse health effects in humans, such as neurodevelopmental deficits, cancer, thyroid hormone disruption, dysfunction of reproductive system, and infertility. Many PBDEs have been listed as chemicals of international concern under the Stockholm Convention on Persistent Organic Pollutants. In this study, the aim was to investigate the structural interactions of PBDEs against thyroid hormone receptor (TRα) with potential implications in reproductive function. Structural binding of four PBDEs, i.e., BDE-28, BDE-100, BDE-153 and BDE-154 was investigated against the ligand binding pocket of TRα using Schrodinger’s induced fit docking, followed by molecular interaction analysis and the binding energy estimation. The results indicated the stable and tight binding of all four PDBE ligands and similarity in the binding interaction pattern to that of TRα native ligand, triiodothyronine (T3). The estimated binding energy value for BDE-153 was the highest among four PBDEs and was more than that of T3. This was followed by BDE-154, which is approximately the same as that of TRα native ligand, T3. Furthermore, the value estimated for BDE-28 was the lowest; however, the binding energy value for BDE-100 was more than BDE-28 and close to that of TRα native ligand, T3. In conclusion, the results of our study suggested the thyroid signaling disruption potential of indicated ligands according to their binding energy order, which can possibly lead to disruption of reproductive function and infertility.

## 1. Introduction

Endocrine-disrupting chemicals (EDCs) are exogenous compounds that interfere with normal functions of the endocrine system, resulting in various endocrine disorders, such as metabolic diseases, neuroendocrine dysfunctions, cancers, reproductive abnormalities, etc. [1,2,3]. The adverse effects can occur through EDC actions at multiple stages in an endocrine system, and hence various mechanisms of action have been reported. These include stimulatory or inhibitory binding to a hormone receptor, inhibition or stimulation of endogenous hormone production, inhibition or stimulation of hormone receptor expression or epigenetic effects [4,5]. Nevertheless, all the indicated mechanisms are expected to disrupt the normal functioning of an endocrine system [4,5]. According to a Health and Environment Alliance (HEAL) report, the proportion of diseases that could be attributed to EDCs is assumed to be 2–5% [6]. According to an estimate for European Union, more than 20% of EDC causation was attributed to diseases, such as IQ loss and associated intellectual problems, autism, attention-deficit hyperactivity disorder, childhood obesity, adult obesity, adult diabetes, cryptorchidism, male infertility, and mortality associated with reduced testosterone, resulting in an economic burden of 209 billion US dollars [7]. Hypothalamic-pituitary-thyroid (HPT) axis is one of the important potential targets of endocrine disruption. The HPT axis is a critical endocrine signaling system in the body responsible for regulating metabolic, growth, and developmental processes [8]. The main end-effector hormones of the HPT axis are thyroxine (T4) and biologically active triiodothyronine (T3). The thyroid hormones are critical for the function of almost all the cells and organ systems in the body, including the heart, nervous system, bone, gastrointestinal tract, musculoskeletal system, reproductive system, etc. [9,10]. For our purpose, the role of thyroid hormones in human reproduction and fertility will be the focus of the discussion to have a better understanding of the mechanism. In this regard, thyroid hormones play a significant role in human reproductive physiology and fertility [9,11,12,13,14]. Briefly, in men, thyroid hormones through their cognate receptor have critical effects on the development of the testis, regulation of spermatogenesis and steroidogenesis, secretion of gonadal (testosterone) and pituitary (FSH and LH) reproductive hormones, and fertility. In addition to affecting Leydig and Sertoli cells, thyroid hormones also have effects on germ cells [15]. In addition, thyroid hormones have effects on epididymis and other accessory sex organs and are important for normal morphology and quality of spermatozoa and their transport in the male reproductive system. Thyroid hormones are also important for maintaining a healthy male libido and for ensuring that the body has enough energy to complete the process of reproduction. Thyroid hormone problems during gestational and neonatal periods have been reported to have a profound adverse effect later on spermatogenesis and male reproductive function [12]. Men with impaired thyroid function have a lower sperm count, lower levels of testosterone, and higher incidences (59% in thyroid dysfunction vs. 30% control) of erectile and ejaculatory dysfunction [11,12,16]. In women, thyroid hormones are vital to the proper functioning of the reproductive system as a primary regulator of metabolism and development of ovarian, uterine, and placental tissues [9,11,17]. Women who have thyroid dysfunction experience several physiological and behavioral changes, including reproductive disorders leading to irregular periods, ovulation problems, fertility issues, miscarriage, premature birth, and low neonatal birth weight [17]. Hence, regarding EDCs, any homeostatic imbalance in thyroid function due to EDCs has the potential to result in reproductive problems and infertility in men and women as indicated above. Polybrominated diphenyl ethers (PBDEs) are a group of most used brominated flame retardants with extensive application in various consumer products to prevent the spread of fire [18]. However, epidemiological studies have suggested that PBDEs act as EDCs, can interfere with several biological processes, such as neurodevelopmental function and cognitive development, thyroid hormone action, and reproductive development and function, and may even be associated with cancer development, etc. [19,20,21,22,23,24,25]. In total, 209 possible different congeners of PBDEs have been reported based on the position and number of the bromine atoms attached to phenyl ring [26,27]. Nevertheless, PBDEs are used as additives and are non-covalently linked to the polymer matrix. Therefore, they can easily leach out from these products into the environment and are hence ubiquitously present in the air, animals, food, and indoor and outdoor environment [27]. They enter the human body through various routes; however, the main routes of PBDEs exposure in humans are contaminated food ingestion followed by contaminated dust inhalation [28,29,30,31,32,33,34,35,36]. PBDEs were listed as Persistent Organic Pollutants (POPs) by the Stockholm Convention in 2009 and 2017 [26]. Therefore, restrictions have been imposed on their use in Europe and the United States, resulting in their phase-out from the market. First, the penta-BDEs and octa-BDEs commercial mixtures were banned from North America and European Union in 2004. Later on, the restrictions on the deca-BDE mixture were placed in 2019; the deca-BDE mixture is only allowed for the production of aeronautics and automobile spare parts [37,38,39]. Nevertheless, PBDEs continue to be a serious challenge in many countries because of their constant release from PBDE-containing products [40,41]. It is estimated that PBDEs will continue to discharge into the environment from in-use stock products and waste until 2050 [42]. Furthermore, many countries have not restricted the use of PBDEs and are still extensively manufacturing and using PBDEs for commercial products [43,44]. In addition to the manufacturing and use, the recycling of PBDEs containing products [45] and the environmental stability of PBDEs [46] also contribute to their ubiquitous environmental presence. Numerous studies have reported the association of PBDE exposure with adverse health outcomes. The economic burden associated with PBDE exposure and associated health outcomes is significant. According to the latest reports, the economic cost of EDC exposure in Canada was CAD 24.6 billion, and the main driver for costs was PBDE-associated health abnormalities amounting to CAD 11.5 billion [47]. Similarly, this cost was USD 340 billion in the United States, and again the major portion of the cost amount was related to PBDE exposure amounting to USD 266 billion. Likewise, in Europe, the economic cost was estimated to amount to USD 217 billion, and USD 12.6 billion was associated with PBDE exposure [48].

PBDEs share their chemical structural resemblance with thyroid hormones, and hence have the potential to interfere with thyroid hormone action. The possible mechanisms include competitive binding with thyroid hormone receptors (TRα) and thyroid hormone transport proteins [49,50,51,52] and an increase in metabolism and clearance of T4 [53]. Numerous studies have reported that PBDEs interfere with T4 transport causing thyroid dysfunction [50,54,55]. In addition, PBDE exposure was associated with adverse effects on penile length, sex hormone binding globulin, testosterone concentrations [56], and shorter anogenital distance [57]. In addition, human studies have reported an inverse association between sperm mobility and PBDE exposure [58]. Furthermore, a detailed review of the literature on the mechanisms of PBDE-induced male reproductive toxicity is also presented [59]. PBDE exposure in adult male rats has shown abnormal reproductive organ weight and sperm count [60,61].

Some of the ubiquitously detected PBDEs are BDE-28, BDE-100, BDE-153, and BDE-154. Perusal of the literature indicated that in general very limited studies are available on the structural binding aspects of PBDEs with TRα. In addition, in silico structural binding characterization of TRα with BDE-28, BDE-100, BDE-153, and BDE-154 has not been reported to the best of our knowledge. Furthermore, the comparative analysis of structural binding parameters and the potential thyroid dysfunction activity as discussed above indicates all four PBDE ligands have not been reported. Therefore, the present study was performed to investigate the structural binding characterization of PBDEs: BDE-28, BDE-100, BDE-153, and BDE-154 against the ligand binding pocket of TRα. The induced fit docking approach was used to perform this docking simulation study. The overall aim of this study was to investigate the potential thyroid dysfunction activity of aforementioned PBDEs with implications for reproductive function and infertility.

## 2. Results

**Figure 1:**
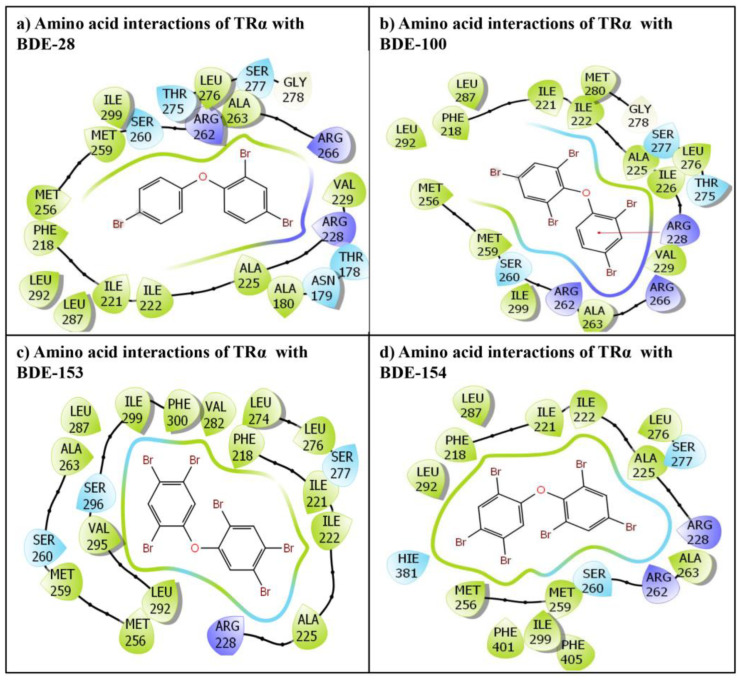
The molecular interactions of PBDE flame retardants (**a**) BDE-28, (**b**) BDE-100, (**c**) BDE-153 and (**d**) BDE-154 with amino acid residues in the TRα ligand binding pocket.

The best-chosen poses for all the indicated ligands, BDE-28, BDE-100, BDE-153, BDE-154, and the TRα native ligand, T3, were carried forward for further analysis. The final chosen poses for all the ligands exhibiting molecular interaction of amino acid residues with respective ligands are presented (Figure 1). The BDE-28 displayed interactions with 22 amino acid residues of TRα (Figure 1a). Further, 21 amino acid residues of TRα were observed exhibiting interactions with BDE-100 (Figure 1b). However, 19 amino acid residues of TRα displayed interactions with BDE-153 (Figure 1c), and for BDE-154, the number of amino residues involved in interactions with TRα was 18 (Figure 1d). 

### 2.1. Induced Fit Docking of BDE-28 with Thyroxine Receptor-α

The TRα/*BDE-28* docking complex displayed several amino acid residue interactions of TRα with ligand BDE-28 in the binding pocket. Overall, 22 TRα amino acid residues displayed various molecular interactions, such as hydrophobic, hydrogen bonding, van der Waals interactions, etc., with the ligand BDE-28. The amino acid residues involved in various interactions were Thr-178, Asn-179, Ala-180, Phe-218, Ile-221, Il2-222, Ala-225, Arg-228, Val-229, Met-256, Met-259, Ser-260, Arg-262, Ala-263, Arg-266, Thr-275, Leu-276, Ser-277, Gly-278, Leu-287, Leu-292, and Ile-299 (Figure 1a). 

**Figure 2:**
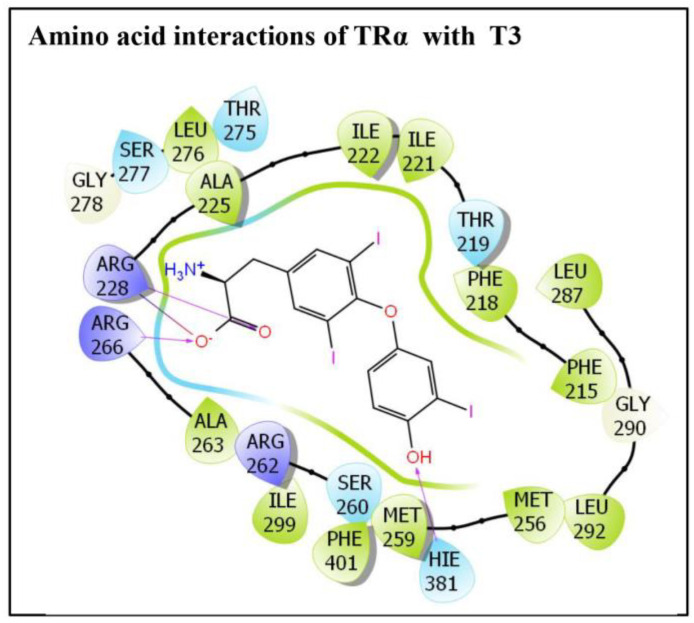
The molecular interactions of a TRα amino acid residues lining the ligand binding pocket with its native ligand, triiodothyronine (T3).

Similarly, the molecular interaction of TRα native ligand, T3, with TRα amino acid residues is also presented (Figure 2). Altogether, 23 TRα amino acid residues displayed various molecular interactions with ligand T3, i.e., Phe-215, Phe-218, Thr-219, Ile-221, Il2-222, Ala-225, Arg-228, Met-256, Met-259, Ser-260, Arg-262, Ala-263, Arg-266, Thr-275, Leu-276, Ser-277, Gly-278, Leu-287, Gly-290, Leu-292, Ile-199, His-381, and Phe-401. In addition, three amino acid residues Arg-228, Arg-266 and His-381 each exhibited one hydrogen bonding interaction with ligand native ligand, T3. In addition, Arg-228 also displayed one salt bridge interaction with the native ligand (Figure 2).

The other parameters such as IFD, Dock score, and Glide score, essential for structural binding analysis and characterization of BDE-28 and TRα native ligand, T3, are presented (Table 1). In addition, another important parameter essential for analysis is binding energy, the estimated binding energy values are also presented (Table 1). However, the estimated binding energy value for TRα native ligand, T3, is more than that of BDE-28. Furthermore, the commonality of the TRα interacting amino acid residues between the TRα-T3 and TRα-BDE28 docking complexes was approximately 82%. However, four more residues i.e., Thr-178, Asn-179, Ala-180, and Val-229, were also observed interacting with TRα/BDE-28 but were absent in the native ligand (TRα-*T3*) complex. 

### 2.2. Induced Fit Docking of BDE-100 with Thyroxine Receptor-α

The docking display pose of *BDE-100* exhibited 21 amino acid residues engaged in various molecular interactions with TRα (Figure 1b). Furthermore, the comparison between the docking poses of the native ligand, T3, and BDE-100 revealed approximately 86% overlap in amino acid interactions. However, several other molecular interactions were also observed in the TRα-BDE100 complex due to additional amino acid residues i.e., Ile-226, Val-229, and Met-280 (Figure 1b). Furthermore, one pi-cation interaction was also displayed by Arg-228.

### 2.3. Induced Fit Docking of BDE-153 Ligand with Thyroxine Recepotor-α

The docking display pose of BDE-153 exhibited 19 amino acid residues engaged in various molecular interactions with TRα (Figure 1c). Furthermore, the comparison between the docking poses of the native ligand, T3, and BDE-153 revealed approximately 74% overlap in amino acid interactions. However, several other molecular interactions were also observed in BDE-153/TRα complex due to additional amino acid residues i.e., Leu-274, Val-282, Val-295, Ser-296, and Phe-300 (Figure 1c).

### 2.4. Induced Fit Docking of BDE-154 Ligand with Thyroxine Receptor-α

The docking display pose of BDE-154 exhibited 18 amino acid residues engaged in various molecular interactions with TRα (Figure 1d). Furthermore, the comparison between the docking poses of the native ligand, T3, and BDE-154 revealed approximately 94% overlap in amino acid interactions. However, one more molecular interaction was also observed in TRα/BDE-154 complex due to additional amino acid residue i.e., Phe-405 (Figure 1d). 

## 3. Discussion

Briefly, the overall results of the IFD experiment suggested the successful and stable placement of all the indicated PBDE flame retardant ligands in the TRα ligand binding pocket. Furthermore, the IFD results also revealed that the structural binding parameters such as IFD score, Docking score, and Glide score estimated for all four ligands were similar to the values estimated for TRα native ligand, T3. In addition, the comparative analysis of the molecular interactions displayed by TRα amino acid residues with all the indicated four PBDEs ligands also showed similarity with the molecular interactions displayed with the TRα native ligand, T3. Furthermore, BDE-153 showed the highest estimated binding energy value among all the ligands, which was even higher than the value calculated for TRα native ligand, T3. This was followed by BDE-154, with an estimated binding energy value approximately similar to that of the TRα native ligand, T3. On contrary, the binding energy value calculated for BDE-28 was the lowest among all the indicated ligands. However, the binding energy value calculated for BDE-100 was also lower but close to the value estimated for TRα native ligand, T3. The overall similarity in structural binding characterization parameters and the docking results between the TRα native ligand, T3, and all four indicated PBDE ligands suggested the resemblance in their binding pattern and position. Therefore, the results predict a potential interference in thyroid hormone signaling by these ligands. The thyroid disruption could further possibly lead to abnormal reproductive function and infertility. Earlier studies have indicated that disruptive thyroid hormone signaling subsequently affects normal reproductive functions such as changes in sex hormone levels, semen quality, spermatogenesis, and erectile abnormalities [12,62]. The potential adverse impact for BDE-153 and BDE-154 seems to be more than for BDE-28 and BDE-100.

In general, very limited studies are available on the structural binding aspects of PBDEs with TRα, and in particular, with the four indicated PBDEs ligands: BDE-28, BDE-100, BDE-153, and BDE-154. However, several in silico studies have reported the potential endocrine-disrupting activity of various PBDE congeners or their structural analogues with other different receptors or transport proteins. For example, Li et al. reported the docking studies of PBDE structural analogues (hydroxylated PBDEs) with TR [63]. Similarly, in our previous study, we reported that BDE-153 structurally interacts with thyroxine binding globulin (TBG) [52]. In another study, we reported the binding of BDE-47, BDE-99, and their structural analogues with TBG [51]. In addition, in vitro studies also indicated PBDEs and their structural analogues compete in binding with TRs and disrupt the thyroid hormone function [63,64]. 

Several epidemiological studies in humans also suggested impaired thyroid hormone activity due to PBDE exposure. The impaired thyroid hormone activity is indicated by altered T3 and T4 levels [65]. In this regard, the association between maternal and cord sera thyroid hormones and PBDEs was observed [19,21,22,23,24,66,67,68,69,70]. Likewise, the PBDEs showed an association with both higher and lower levels of T3 in pregnant women [22,23]. Several studies have reported an inverse association between the total T3 (TT3) and BDE-153 in pregnant women during the first trimester [21,71]. Another study in human newborns indicated a negative association for prenatal PBDE exposure with total T4 (TT4) and free T4 (FT4) levels in cord blood [72]. A study performed on 260 Canadian pregnant women reported an increase in FT4 concentrations with BDE-47 exposure during the first trimester; however, TT4 showed an inverse association [24]. A study indicated a 4–8% increase in maternal total and free T3 and T4 levels with 10-fold increases in BDE-28 and -47 [73]. Another study reported higher levels of maternal TT4 with a 10-fold increase in BDE-47 concentration [22]. In addition, the hyperthyroid effect was also observed in the human non-pregnant cohort [20,74,75]. A study on women samples from the United States suggested an association between PBDE exposure and thyroid diseases. Furthermore, the observed effects were greater in post-menopause women suggesting that altered estrogen levels in menopause may enhance thyroid signaling disruption by PBDEs [27]. In addition, studies conducted on men have also shown positive associations between PBDE exposure and TT4 and FT4 levels [20,75]. 

Similarly, laboratory animal studies have also reported altered thyroid hormone levels resulting from PBDE exposure [76]. In this regard, altered T4 levels have been reported on PBDE exposure in rodents, felines, and birds [65,77,78]. However, the majority of the animal studies on rodents indicated reduced serum T4 levels on PBDE exposure causing hypothyroidism [49,53]. The studies on rats reported that PBDEs bind with TBG [79], and an inverse association was observed between PBDE exposure and thyroid hormone levels [80]. Likewise, decreased T4 levels were reported in rat offspring subjected to PBDE exposure during the gestational period [81]. A study reported a significant decrease in T4 levels in pre and postnatally PBDEs exposed rodents [54,82]. A study on zebrafish aimed to understand the transgenerational risks of BDE-209 exposure reported decreased T4 levels and downregulation in the thyroid hormone receptor gene in F1 individuals [83]. Thyroid hormones have a critical role in the human male and female reproductive system including male reproductive tract development, spermatogenesis, and male fertility [84]. Any interference in thyroid function is a potential cause of male reproductive dysfunction and infertility. In regard to the current study, the results show that thyroid signaling disruption potential of PBDEs is a potential indication of subsequent adverse effects on male fertility. This is further reinforced by human studies that have directly shown an association of PBDEs with adverse effects on reproductive function [85]. In this regard, BDE-153 exposure was associated with adverse effects on sperm concentration and testes volume [86]. Similarly, higher serum levels of BDE-154 were associated with adverse effects on testes volume, sex hormone binding globulin, testosterone concentrations, and penile length [56]. Likewise, another study indicated that BDE-100 exposure was associated with abnormal sex hormone concentrations [20]. A study on a Shanghai (China) birth cohort indicated that prenatal exposure to BDE-100, BDE-153, and other PBDEs in boys was associated with shorter anogenital distance [57]. Previous studies on other PBDEs have also associated their prenatal exposure with disturbed reproductive tract development in infants [56]. Two studies have reviewed the available literature on the reproductive toxicity caused by PBDEs in humans and animals [34,87]. In laboratory animal studies, prenatal exposure to PBDEs (BDE-47, BDE-99, and DE-71) in rats and mice was reported to cause changes in the relative weight of the testis and epididymis. The compounds were also reported to affect the production of sperm [88]. In another study, postnatal exposure to BDE-209 in male mice affected sperm motion and motility [89]. In addition, exposure to the penta-BDE mixture [61] or deca-BDE [90] in adult male rats resulted in a decrease in the weight of epididymis and seminal vesicle along with a decrease in serum T4 levels. A study in adult male rats suggested that prenatal BDE-47 exposure at environmentally relevant doses causes dysregulation in histone–protamine exchange during spermatogenesis and can potentially result in an aberrant sperm epigenome [91]. Nevertheless, this study is of significant relevance in assessing the impact of environmental toxicants on reproductive outcomes in couples seeking fertility treatment. This study, however, has several limitations. First, this study is a molecular docking simulation study that in itself has its own limitations. Therefore, studies using in vitro and in vivo model systems are suggested to confirm the results. Secondly, our study is an indirect extrapolation of thyroid disruption effects of PBDEs on reproductive function. Therefore, further direct in silico, in vitro, and in vivo studies are suggested to evaluate the association of PBDEs with reproductive dysfunction. The integrated systems biology approach using various model systems is required to overcome these limitations and gain deeper insights into the effects of PBDE on reproductive dysfunction and fertility. 

## 4. Materials and Methods

The three-dimensional structural coordinates of PBDE flame retardants: BDE-28, BDE-100, BDE-153, and BDE-154, were downloaded from the PubChem compound database (https://pubchem.ncbi.nlm.nih.gov/) on 10 June 2021. The aforementioned ligands were chosen as they are very commonly used PBDEs and are detected in a large section of the population [92]. It was followed by structural binding characterization of these ligands using Schrodinger 2017 suite with Maestro 11.4 as a graphical user interface (Schrodinger, LLC, New York, NY, USA, 2017). The detailed methodology is described in our previous studies [93,94].

### 4.1. Protein Preparation

The Protein Data Bank (PDB; http://www.rcsb.org/) was searched on 11 June 2021, and three-dimensional structural coordinates solved at 1.87 Å resolution for the crystal complex of ligand T3 with TRα (PDB code: 2H79) were retrieved. The retrieved coordinates were imported to Glide docking software, and the protein crystal complex was subjected to further processing and prepared for docking studies using the protein preparation wizard workflow of Schrodinger Glide (Schrodinger suite 2017-4; Schrodinger, LLC) as described previously [93,94]. Briefly, the missing hydrogen atoms and charges were added, and water molecules were removed from the crystal complex structure. The metal ionization states were corrected, and the bond order to HET groups was also enumerated. In addition, the N-terminal and the C-terminal of the protein were capped with ACE (N-acetyl) and NMA (N-methyl amide), respectively. The amino acid residues of the protein receptor with multiple occupancies or missing atoms were also highlighted during this step. It was followed by hydrogen bond network optimization by means of a systematic, cluster-based approach and energy minimization steps.

### 4.2. Ligand Preparation

**Figure 3:**
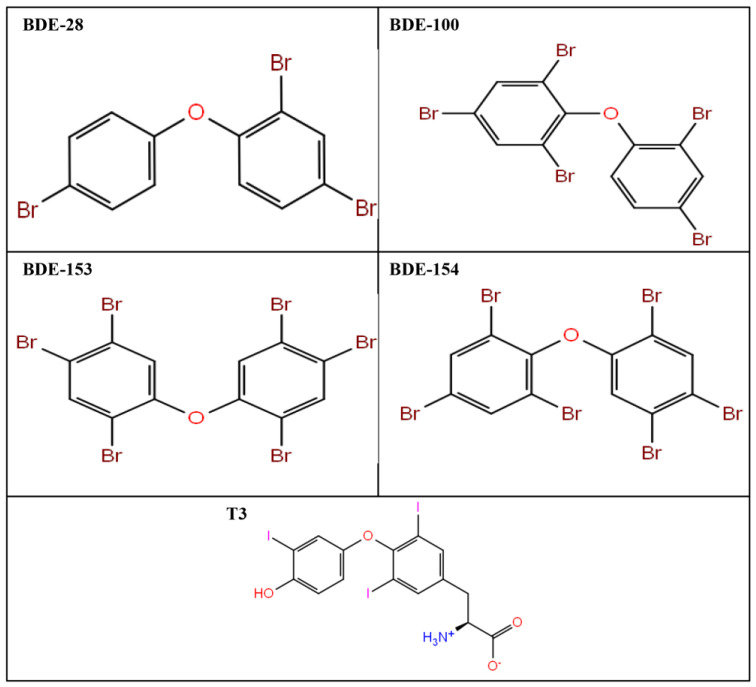
Two-dimensional structure of polybrominated diphenyl ethers (PBDEs) flame retardants: BDE-28, BDE-100, BDE-153, BDE-154, and a TRα native ligand, T3.

The three-dimensional structural coordinates for commonly used PBDE flame retardants: BDE-28, BDE-100, BDE-153, and BDE-154, were downloaded from the PubChem compound database (https://pubchem.ncbi.nlm.nih.gov/) on 10 June 2021. The PubChem compound identity for all the PBDE ligands is mentioned in Table 1. These ligands were processed and prepared for simulation studies by the LigPrep module of Schrodinger (Schrodinger 2017: LigPrep, Schrodinger, LLC, New York, NY, USA), which is a very efficient module preparing approximately one ligand per second for further computational processes. The Ligprep module corrects the Lewis structure and also the mistakes in the ligand. It produces an accurate three-dimensional structure of the ligand following the minimization step. Furthermore, the structures that fail to meet user-specified criteria are removed using the filter. In addition, various possible ring conformations, stereoisomers, tautomeric and ionization states are also generated from the given input ligand structure. The two-dimensional structures of BDE-28, BDE-100, BDE-153, and BDE-154 are presented in Figure 3.

### 4.3. Induced Fit Docking

The Schrodinger’s Induced Fit Docking (IFD) module was employed to perform the docking of TRα native ligand, T3, and commonly used PBDE flame retardants: BDE-28, BDE-100, BDE-153, and BDE-154 in the TRα ligand binding pocket as described in detail previously [93,94]. Briefly, we first generated a grid at TRα native ligand, T3, binding site. It was followed by constrained minimization of TRα using the protein preparation step. The IFD induces flexibility in both the ligand as well as ligand binding pocket of the protein receptor and does not adopt a rigid docking approach. Initial Glide docking was performed using a softened potential and optional side chain removal for all the ligands, and by default, twenty docking poses were retained. The side chains in amino acids were predicted followed by energy minimization for receptor as well as ligand in each pose. It was followed by Glide re-docking and IFD score estimation. Likewise, an extended sampling protocol was also performed. All four indicated ligands exhibited successful docking in the TRα ligand binding pocket and were placed stably in the TRα ligand binding pocket following IFD, indicating their stable binding. The IFD approach generated several docking poses for each ligand; however, only the best poses were chosen and carried forward for structural characterization. Likewise, the IFD was also performed on TRα native ligand, T3.

### 4.4. Binding Affinity Calculations

The binding affinity of BDE-28, BDE-100, BDE-153, and BDE-154 for TRα ligand binding pocket was estimated using the MMGB-SA function in the Prime module of Schrodinger 2017 as described previously [93,94]. The estimated binding energy (ΔGBind) values indicate how strongly ligands are bound in the ligand binding pocket. The following equation is used to estimate the binding free energy (Prime MMGBSA DG bind):

ΔG_bind_ = E_complex_(minimized) − [E_ligand_(minimized) + E_receptor_(minimized)].

where ΔG_Bind_ is binding free energy, and E_complex_ (minimized, E_ligand_(minimized), and E_receptor_(minimized) are minimized energies of receptor-ligand complex, ligand and receptor respectively.

## 5. Conclusions

In conclusion, the results of this study suggested that among all the indicated four ligands, BDE-153 and BDE-154 have the higher potential than BDE-100 to interfere with thyroid hormone signaling and impair thyroid function with subsequent adverse implication for reproductive function and fertility. The results also suggested the least thyroid signaling disrupting potential for BDE-28. However, further studies are warranted to enhance the understanding of the role of these PBDE flame retardants in impaired thyroid and reproductive function and associated adverse health effects. 

## Figures and Tables

**Table 1:** Structural binding indices of polybrominated diphenyl ethers (PBDEs) flame retardants; BDE-28, BDE-100, BDE-153, BDE-154 and a TRα native ligand, T3.

Ligand	Number of Interacting TRα Residues	Percentage of Interacting Residues Common with Native Ligand (%)	IFD Score	Docking Score (Kcal/mol)	Glide Score (Kcal/mol)	MMGB-SA (Kcal/mol)
BDE-28	22	81.81	−558.04	−7.24	−7.24	−93.19
BDE-100	21	85.75	−558.16	−7.03	−7.03	−114.81
BDE-153	19	73.68	−560.99	−8.78	−8.78	−146.89
BDE-154	18	94.44	−561.93	−8.85	−8.85	−133.29
T3	23	100	−564.42	−9.44	−9.44	−133.53

## Data Availability

Not applicable. All data is contained within the article.
